# Male Homosexual Preference: Where, When, Why?

**DOI:** 10.1371/journal.pone.0134817

**Published:** 2015-08-12

**Authors:** Julien Barthes, Pierre-André Crochet, Michel Raymond

**Affiliations:** 1 Human Evolutionary Biology Team, University of Montpellier, Montpellier, France; 2 CNRS-UMR 5175, Centre d'Ecologie Fonctionnelle et Evolutive, Montpellier, France; 3 CNRS-UMR 5554, Institute of Evolutionary Sciences, Montpellier, France; The University of New South Wales, AUSTRALIA

## Abstract

Male homosexual preference (MHP) has long been of interest to scholars studying the evolution of human sexuality. Indeed, MHP is partially heritable, induces a reproductive cost and is common. MHP has thus been considered a Darwinian paradox. Several questions arise when MHP is considered in an evolutionary context. At what point did MHP appear in the human evolutionary history? Is MHP present in all human groups? How has MHP evolved, given that MHP is a reproductively costly trait? These questions were addressed here, using data from the anthropological and archaeological literature. Our detailed analysis of the available data challenges the common view of MHP being a “virtually universal” trait present in humans since prehistory. The conditions under which it is possible to affirm that MHP was present in past societies are discussed. Furthermore, using anthropological reports, the presence or absence of MHP was documented for 107 societies, allowing us to conclude that evidence of the absence of MHP is available for some societies. A recent evolutionary hypothesis has argued that social stratification together with hypergyny (the hypergyny hypothesis) are necessary conditions for the evolution of MHP. Here, the link between the level of stratification and the probability of observing MHP was tested using an unprecedented large dataset. Furthermore, the test was performed for the first time by controlling for the phylogenetic non-independence between societies. A positive relationship was observed between the level of social stratification and the probability of observing MHP, supporting the hypergyny hypothesis.

## Introduction

Male homosexual preference (MHP), sexual attraction to male partners even if female partners are available, is an evolutionary enigma because, in humans, preference for male-male relationships is partially heritable [1, 2], imposes a fertility cost (lower offspring number) [3–5] and is relatively common in some societies (2%–6% in Western countries) for such a costly trait [6]. The origin and maintenance of MHP in humans has long been a matter of interest [7].

When did MHP first arise? Male homosexual behavior (MHB) has been described in nearly 450 animal species, although these behaviors appear to be socially induced (context dependent), for example through the lack of accessible female partners, during intrasexual conflict or as “social glue” [8–10]. Socially induced homosexual behavior has also been documented in humans, for example when women are not available [11, 12] or in a ritualized form [13]. MHP has also been described in domesticated sheep [14, 15], suggesting that homosexual preference could be (at least indirectly) selected for. No clear cases of MHP have been documented in any non-human species outside of undisturbed social environments (i.e., zoos or domestic species) [8, 9]. Apparently, MHP appears to be restricted to humans.

When did MHP appear during the course of human evolution? Many authors have suggested that MHP dates back to prehistoric [16–19] or early historic time [20], although archaeological evidence in support of such claims is questionable, and in some cases, the validity of the supporting evidence has been challenged [21].

Cross-cultural studies suggest that MHP is widespread among ethnic groups, although the number of societies studied in details for this trait is rather limited e.g., [20, 22, 23]. However, a number of recent reports of the absence of homosexual behavior (and thus of MHP) in some ethnic groups [24] has questioned the idea that MHP is virtually universal. This variation in the presence and absence of MHP among ethnic groups remains to be documented.

From an evolutionary point of view, the emergence and maintenance of homosexual preferences require that the decrease in fertility associated with MHP should be compensated by sufficient increases in fertility among close relatives. This increase may be promoted behaviorally by kin selection [25, 26], although empirical evidence is not always consistent. In Western societies, no difference between men with a MHP and heterosexual men is observed in the desire to invest in nieces and nephews [27, 28]; in Samoa, it has been observed that the Fa'afafine (the third gender associated with a MHP) invest more in their nieces and nephews than heterosexual men [18, 25, 29, 30]. These conflicting sources of evidence demand further research.

Alternatively, the increase in fertility in a close relative could be the result of an antagonistic factor. A sexually antagonistic gene that favors MHP in males and that increases fecundity in females has been proposed [31]. Several studies support this hypothesis [4, 22, 31–34] and other have provided results that are consistent with predictions from this hypothesis [22, 34–38]. However why such an effect would not operate also in wild animals is unclear. Sexually antagonistic genes are either fixed (when the advantage is higher than the cost) or selected against (when the cost is higher than the advantage). When the frequency of a sexually antagonistic gene increases, selection to decrease the cost could eventually operate (for example through the selection of a modifier gene), thus decreasing the fixation time of the antagonistic gene. In any case, such sexually antagonistic genes are only transiently observed in natural population, perhaps explaining the absence–so far–of reports of homosexual preference in wild animals. A recent change in social conditions could change the relative fitness advantage and cost of such gene, thus enhancing its selection.

It has been recently proposed that selection for such sexually antagonistic genes could be promoted in social contexts specific to some human societies, where there is social stratification and hypergyny (i.e., a bride marries a groom of higher social status) [39]. Indeed in a stratified society, populations are organized into different groups (or classes) in which people share similar socioeconomic conditions. These groups can be ranked hierarchically depending on their access to resources (with more resources for the top class). This social inequality also affects the expected reproductive success of each group (with higher reproductive success associated with the top class) [40–45]. This hypergyny hypothesis posits that females carrying the sexual antagonistic variant (associated with MHP in males) will signal increased levels of fertility (through higher femininity or attractiveness), thereby increasing their probability of reproducing in a wealthier social environment. Such a sexually antagonistic gene will then provide a direct advantage (by increasing fertility) and an indirect advantage (by increasing the probability of marrying into higher social classes). Such a process may promote MHP in stratified societies, and indeed, a comparative analysis suggests that social stratification level is a predictor of the presence of MHP in a society [39]. Several potential confounding variables were considered in this analysis, with the conclusion that none of them significantly influenced the probability of report of homosexual preferences. These variables included population density (a good proxy of the number of indigenous people met by the anthropologist), geographical location and presence of moralizing gods. However, this comparative study considered only 48 societies, and phylogenetic dependence among them (Galton's problem) [46] was not clearly addressed.

While the questions of where, when and why MHP were usually considered separately, here we argue that it is particularly important to address these questions all together with an evolutionary perspective. Indeed, the information needed to answer each question sheds light on the others. We will review the archaeological literature usually cited as evidence of MHP, and analyze the distribution of MHP among current human populations. A comparative analysis on a large number of societies will then be performed to test the hypergyny hypothesis, while correcting for the phylogenetic relationships among the human societies.

## Materials and Methods

### Archaeological materials

Archaeological data that have been repeatedly cited as evidence for the existence of MHP during prehistoric and early historic times were gathered from scientific papers [47] and books [16, 19, 48] (Table 1). Data originating from unpublished sources (such as media reports) were not considered. A specialist of the post-paleolithic parietal Levantine art in Spain, A. Grimal Navarro [49] was contacted concerning statements from paintings from one Spanish cave, and his comments were cited as personal communication.

**Table 1:** Archaeological data often cited as evidence of MHP in prehistoric societies.

Country	Ethnic group	Date	Type	Place	Description	Criticism
Norway	European	Mesolithic (before written texts)	Rock engraving	Bardal Panel	Two human figures locked in "rear-entry" sexual intercourse [16]	Inconsistencies in the details of the different plates representing the scene. No definitive evidence of penetration. The sex of the supposedly penetrated individual is not determined.
Spain	European	Mesolithic (before written texts)	Rock engraving	Cuevas de la vieja panel. Albacete	Smaller male performs a fellatio to the "dominant" male [16]	The two figures have been drawn at different time, using different techniques and probably do not belong to the same scene (A. Grimmal Navarro, Comm. pers.)
Sweden	European	Mesolithic (before written texts)	Rock engraving	Hoghem in Tanum	"It is [. . .] possible that both figures are male–this, of course would require us to suspend our prejudices about what such scenes would then mean, and from my experience it is clear that most archaeologist are unwilling or unable to do so, and will go to extraordinary lengths to hang on to the heterosexual hypothesis" [19]	No direct evidence of homosexual relationship. The nature of the relation between the two figures and the sex of the protagonist are not identifiable.
Peru	Moche	200 BCE-600 CE(before written texts)	At least four vases	Peru	"Homosexual acts between males are found on at least four cases [. . .] each showing consensual anal intercourse" [48]	"It is difficult not only to attribute precise dates and provenance but to assign valid and convincing interpretation and to attach meaning (not only their meaning for us, but their possible meaning for the Moche culture itself) to these objects, since we have no text to interpret them" [48]
Egypt	Egyptian	2400 BCE (early Historic)	Egyptian grave	Necropolis of Saqqara	"Whatever the biological relationship may have been between Niankhkhnum and Khnumhotep, their iconography vocabulary was most closely aligned to that used to portray conjugal sentiment between husband and wife." [47]	"Since the embracing and handholding scenes are unique in private tombs, little can be said about their meaning beyond the fact that they express publicly the close involvement of the two men" (Baines 1985 in Reeder, 2000). "Altenmtiller and Moussa suggested Niankhkhnum and Khnumhotep were brothers, possibly twin" [47]

See S1 Table for additional details.

### Hypergyny hypothesis

Data on the presence or absence of MHP in different societies have been gathered using existing reviews [6, 50–52] and additional anthropological monographs and studies [25, 53–55]. The large database of anthropological monographs from the Human Relations Area Files (eHRAF) was searched using “homosexuality” as a keyword. The eHRAF allows to browse the original monographs. This is important, as the distinction between MHP and MHB is not apparent in the pre-coded variables of the Standard Cross-Cultural Sample (SCCS). However, while the SCCS was designed to try to address the Galton's problem, using the eHRAF as a source of data require to control for pseudoreplication (see below). Relevant excerpts of the monographs concerning homosexuality have been extracted for each society. Positive clues of the presence of MHP in a society included a description from an anthropologist of individuals displaying a MHP, the existence of a word for MHP, and a description of a third gender including individuals displaying a MHP such as the Fa'afafine of Samoa [25] or the Berdache of North America [56]. Negative clues included the absence of a word and concept for MHP or the direct conclusion from an anthropologist after having explicitly asked for the existence of homosexuality. When a clear distinction between MHP and homosexual behavior could not be made, the case was not considered further. Societies were classified as (1) MHP present, (2) presence of MHP very likely and (3) absence of MHP very likely or (4) MHP absent. Classes (1) and (2) were lumped together, as well as classes (3) and (4), and coded as “MHP very likely” and “MHP very unlikely,” respectively.

Two independent measures of the level of social stratification were gathered to control for the dependence of model sensitivity on the way in which social stratification was rated. First, the “Class stratification” variable of the Ethnographic Atlas (EA) was used [57, 58]. The five factors of this variable have been merged into three levels in order to suppress empty classes (not supported by the statistical method used here, i.e., the ape package in R). The resulting factors were: (1) absence of social stratification (factor 1), (2) simple stratification based on wealth or elite (merging factors 2 and 3) and (3) complex stratification (merging factors 4 and 5). Second, data on the level of social stratification were gathered using eHRAF, relevant anthropological monographs and books [53–55, 59–64]. From the excerpts, the level of stratification was first assessed for each society on a scale ranging from 1 to 5, corresponding to the number of classes that could be identified, and then reduced to 3 levels (see S1 Text): (1) no stratification, (2) moderately stratified, and (3) strongly stratified. The two measures of social stratification were available for 72 societies and were strongly correlated τ = 0.65 (P < 0.0001).

### Statistical analyses

Generalized linear models were used to test the influence of the level of stratification on the probability of observing MHP (coded as 0 or 1). To take into account the non-independence among societies, generalized estimating equations (GEE) were used. GEE allow the relationship between a response variable and explanatory variables in a generalized linear model framework to be analyzed by taking into account a structure of correlation between the items of the response variable [65]. As the exact ancestral relationships among all of the sampled societies were not available, two proxies of the true phylogeny were used in the analysis. First, a linguistic phylogeny, known to parallels genetic trees [66, 67]. Indeed, the use of linguistic trees has been highlighted as a good way to deal with the Galton’s problem in comparative anthropology [68]. Second, the geodesic distance between societies was used as a proxy of their cultural and historical closeness, with the implicit assumption that geographic distances are related to cultural similarities.

A linguistic similarity matrix between societies was extracted from the World language tree of lexical similarity from version 15 of the Automated Similarity Judgement Program database [69–72]. This linguistic phylogeny is based on the 40 more stable words of a Swadesh list [73, 74]. The phylogenetic trees containing only the societies for which data on MHP and stratification were available were extracted from the World language tree of lexical similarity and were used for an additional statistical analysis. The analysis using the linguistic phylogeny to control for pseudoreplication was performed with each of the two distinct measures of the level of social stratification (one extracted from the EA, and one extracted from eHRAF and complementary sources, see above). As the results did not differ qualitatively, further analyses were performed using the largest sample (i.e., the sample based on the EA information on social stratification).

Then, geographic localization of each society was extracted from their latitude and longitude as coded in the Ethnographic Atlas. The great-circle distance between each society was then calculated. The resulting matrix of distances between societies was used to build a tree of distance based on the neighbor-joining method [75], and used in additional statistical analyses. This tree was integrated in an analysis with the presence and absence of MHP as a response variable and social stratification as an explanatory variable.

To compare these results with a previous analysis [39], a classical generalized linear model with binomial error was performed, using the geographic zone as a confounding variable (in that study, density of population had no significant effect and was thus not considered here). Geographic zone was defined using variable V200 of the Standard Cross-Cultural Sample (SCCS), with six modalities: Africa, Circum-Mediterranean, East Eurasia, Insular Pacific, North America, and South America. Presence and absence of MHP was the response variable, the level of stratification was an explanatory variable, and the geographic zone was a possible confounding variable. All analyses were conducted using R version 2.15.2 [76] using the “ape” package version 3.0–7 for GEE [77] and the irr and psych packages for the Kappa coefficient [78].

## Results

### Archaeological evidence for homosexual behavior

Several prehistoric references were examined. The first one corresponds to Mesolithic paintings, the panel of the Cueva de la Vieja (Albacete, Spain) belonging to the Spanish Levantine art. The claim that homosexuality is observed is based on two individuals where “*The head of the smaller male is directed to the erect penis of the central (dominant) male–fellatio is being performed*” [16]. However, a completely distinct interpretation is provided by a specialist of this cave, and more generally of the Levantine art in Spain. About this specific panel, he noted that “*The face of the bottom individual is directed in a direction opposed to the penis*. *Furthermore*, *the color and craftsmanship of this character is distinct*, *he also holds a bow*. *We then can conclude that the two figures have not been made at the same period and are not part of the same scene*” [79]. More generally, G. Nash states that the “*Act of male homosexual buggery*, *masturbation and fellatio and auto-fellatio are present on rock paintings from the Spanish Levant*”; however, A. Grimmal Navarro underscores the following: "*It must be noted that neither in the Levantine art (~10 000 years ago–Mesolithic)*, *to which the Cueva de la Vieja panel belongs*, *nor in the Iberian schematic art (~6500 years ago–Neolithic)*, *can there be found any sexual scenes*, *either being heterosexual or homosexual*" (A. Grimal Navarro, personal communication, 2013; our own translation).

The second refers to a petroglyph of Bardal, Norway, dating back to the Mesolithic period. According to Nash [16], “*Two of the human figures are locked in ‘rear-entry’ sexual intercourse*”, although this interpretation has been challenged, as the sex of the smaller and penetrated individual is not identifiable [21]. When we inspected the depiction of the petroglyphs from the original reports [80–82], we identified several problems due to the variability in drawings. In some, the identification of the smaller individual as a human is questionable, as two lines above the head suggest a horned animal, consistent with hair depicted below the belly. In others, lines suggesting a rear-entry penetration are not reported. This petroglyph could thus represent either homosexual intercourse, heterosexual intercourse, zoophilia, or something else (other references are analyzed in the supporting information section: Archaeological evidences).

### Social stratification & MHP

**Fig 1:**
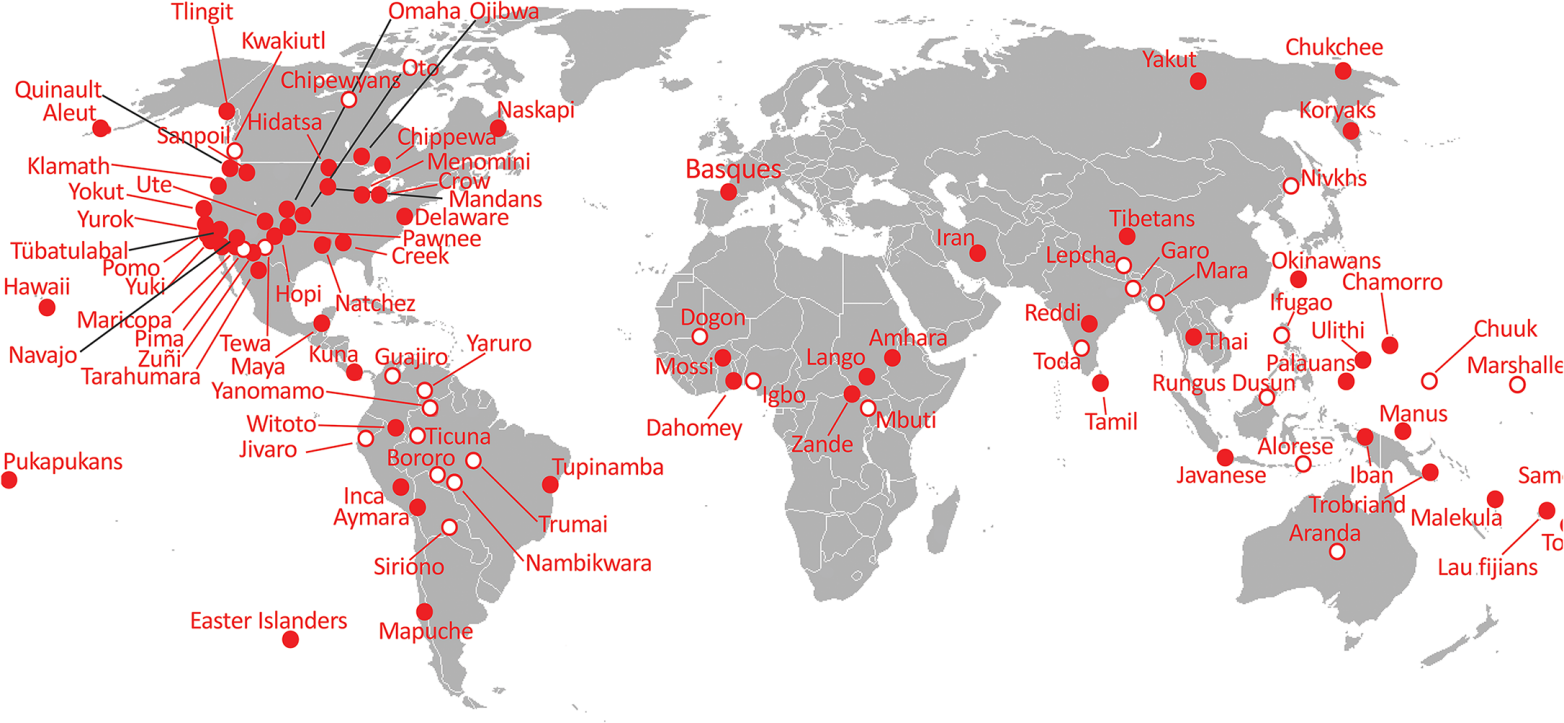
Geographic distribution of the sampled societies (using the EA to assess the level of stratification). Full circles: societies with MHP; empty circles: societies without MHP.

Our aim was to test the link between the presence and absence of MHP (response variable) and the level of social stratification (explanatory variable). First, two models were run using linguistic phylogenies to control for possible pseudoreplication due to common ancestry (Table 2). In the first model, the level of social stratification was estimated using data from eHRAF completed with other sources (see Materials and Methods). The resulting sample included 86 societies (see S1 Fig). The level of social stratification significantly increases the probability of observing MHP (F2, 84 = 21.25, P < 0.0001). The probability of observing MHP was 0.28, 0.75 and 0.91 for non-stratified, moderately stratified and strongly stratified societies, respectively. In the second model, the level of social stratification was estimated using data from the EA database. The final sample represented 92 societies (including 15 not present in the previous sample; see Fig 1). Again, the probability of observing MHP in societies significantly increases with the level of stratification (F_2,90_ = 10.17, P = 0.0001). The probability of observing MHP was 0.48, 0.68 and 0.89 for non-stratified, moderately stratified and strongly stratified societies, respectively. Further analyses were only performed with the largest sample (i.e., data for which social stratification was based on the EA).

**Table 2:** Results of the different models testing the link between the level of stratification and the probability of observing MHP.

Models–data source [pseudoreplication control]	Variables	Estimate	SE	p-value
Model 1—eHRAF [Linguistic phylogeny]	Intercept	-0.93	0.39	
	**Stratification**			<0.0001
	Strat. level 1	2.04	0.61	
	Strat. level 2	3.37	0.84	
Model 2- EA [Linguistic phylogeny]	Intercept	-0.06	0.33	
	**Stratification**			0.0001
	Strat. level 1	0.80	0.54	
	Strat. level 2	2.19	0.70	
Model 3- EA [Geographic phylogeny]	Intercept	0.64	0.33	
	**Stratification**			0.003
	Strat. level 1	0.75	0.54	
	Strat. level 2	1.92	0.70	
Model 4—EA [Geographic origin]	Intercept	-1.33	1.01	
	**Geographic origin**			0.0009
	Cicum-med	16.81	1900	
	East Eurasia	0.06	1.10	
	Insular Pacific	0.11	1.04	
	North America	2.74	1.12	
	South America	0.15	1.08	
	**Stratification**			0.0003
	Strat. level 1	1.54	0.68	
	Strat. level 2	2.90	0.84	

Models 1, 2 and 3 use GEE while model 4 uses GLM.

Another proxy of common ancestry other than linguistic similarity is the geographical distance between societies. The resulting model displayed a significant effect of the level of social stratification on the probability of observing MHP (F_2,90_ = 7.94, P = 0.003). The probability of observing MHP was 0.65, 0.80 and 0.93 for non-stratified, moderately stratified and strongly stratified societies, respectively.

For comparison with a previous study [39], a standard generalized linear model with binomial error was performed using the presence and absence of MHP as a response variable and social stratification as an explanatory variable, while controlling for the geographical zone. The Nagelkerke’s R^2^ for this model is estimated at 0.41. A significant effect of the level of social stratification was observed (X^2^ = 16.33, df = 2, P = 0.0003) together with a significant effect of geographic zone (X^2^ = 20.76, df = 5, P = 0.0009), driven by the high prevalence of MHP among indigenous people of North America. The effect of social stratification remains qualitatively unchanged when the analysis is performed after removing the North American societies.

## Discussion

When MHP is considered from an evolutionary perspective, three main questions arise. At what point did MHP appear in the course of human evolution? Is MHP present in all human groups? Why has this apparently deleterious trait been selected for?

### Archaeology of MHP

It has been claimed repeatedly that MHP dates back to prehistoric time [16–19, 32]. After reviewing in detail the cited evidence, the only conclusion that can be made is that no cited source of evidence has clearly demonstrated the existence of a homosexual preference in a human society. In fact, no cited source can even be considered as an unambiguous demonstration of homosexual behavior (see S1 Text). This conclusion does not preclude the possibility that other prehistoric art or artifacts might be discovered that clearly depict homosexual behavior. This would not be surprising, considering that homosexual behavior is frequently described in great apes [8] and many human cultures [13, 20]. However, depictions of homosexual behavior cannot be used as evidence for the existence of homosexual preferences. Indeed, it seems difficult to devise a robust way of identifying MHP from prehistoric pictorial and sculptural art. It is even more difficult to demonstrate the presence of MHP in a past society without additional information such as written texts.

Only comparative studies in extant traditional societies isolated as much as possible from historical cultures could provide useful insight. It has been claimed recently that MHP existed in “ancestral” humans under the form of “sex-gender discordant” homosexuality [17]. This assertion is based on a cross-cultural analysis of traditional societies that were separated into transgendered and non-transgendered societies. However, this conclusion is grounded on the hypothesis that transgendered societies are ancestral. Additionally, MHP could be present in many non-transgendered societies. Thus, the specific case of transgendered societies cannot be used to make robust inferences to a broader phenomenon.

From archaeological remains, the first persuasive evidence that MHP was possibly present dates from early historic times, from the grave of Khnumhotep and Niankhkhnum in Ancient Egypt (circa 2400 BCE) [47]. Written texts are a precious source of information on sexual preferences and allow the identification of the presence of homosexual behaviors and homosexual preferences in past societies, including ancient Greece, ancient Rome and old China [6, 83, 84].

### Distribution of MHP

Contrary to the widely held view that MHP is present in all contemporary societies e.g., [20, 22], the anthropological data gathered here show that MHP is likely absent from some societies, especially those that display low levels of stratification. Anthropologists that have explicitly searched for signs of MHP have acknowledged its absence: among the Alorese “*The fact is that homosexuality as such is not known either among women or men*” [85]; “*Homosexuality and onanism are unknown among the Bororo*, *as well as among the majority of the Indian tribes visited by me*” [62]; “*Homosexuality is said to be unknown in Ulithi*, *but it is admitted as a possibility*” [86]; among the Ifaluk people “*The people know of no cases of homosexuality or of sexual perversions*, *nor did I observe any*” [87]; and among the Yanomamö, “*Most of the unmarried young men in Bisaasi-tedi were having homosexual relationships with each other [*…*] The men involved in these affairs*, *however*, *were hardly more than teenagers; I have no cases of adult men satisfying their sexual needs by homosexuality*” [11]. The most recent account of the absence of MHP concerns the Aka people, a hunter-gatherer group from Central African Republic for which an anthropologist noted that “*The Aka*, *in particular*, *had a difficult time understanding the concept and mechanics of same sex relationships*. *No word existed and it was necessary to repeatedly describe the sexual act*. *Some mentioned that sometimes children of the same sex (two boys or two girls) imitate parental sex while playing in camp and we have observed these playful interactions*” [24].

Some could argue that anthropologists have failed to detect MHP in some societies because it was negatively sanctioned or taboo. This could be true in some cases; however, as mentioned by [21], these same anthropologists have also described negatively sanctioned behaviors such as murder, theft, infanticide and extramarital affairs. Thus, the documented absence of MHP in some societies, where other taboo behaviors were nevertheless uncovered, suggests that MHP may often be absent. The presence or absence of MHP can thus be variable across ethnic groups.

### Hypergyny hypothesis

A predictor of the presence of MHP in a given society is the level of social stratification. This result remains well supported even when non-independence among societies modelled as linguistic or geographical proximities is accounted for and when two independent measures of stratification level (using the EA or eHRAF) were considered. In all cases, the probability of observing MHP increases with the level of social stratification. It is thus expected that several social variables directly related to stratification would also be associated with MHP. There have been previous attempts to identify social variables related to homosexuality [88]. However, Barber did not distinguished male and female homosexuality, and did not differentiate between homosexual preference (MHP) and behavior, thus his results are not directly comparable to ours. Despite these caveats, Barber found several traits often associated with traditional stratified societies (large community size, agricultural food, low levels of female control over sex), that predict his measure of the homosexual frequency. Unfortunately, he did not studied directly the stratification level.

In stratified societies, MHP is most likely not selected for directly as it represents a fitness cost, but we hypothesize that it is associated with a pleiotropic and antagonist factor. It has been proposed that the fitness advantage of such a pleiotropic factor is an increase in female fertility [31], which would affect the probability of females marrying men from higher social classes in stratified societies [39]. This effect of stratification level on the probability of observing MHP is thus consistent with the hypergyny hypothesis [39]. The present data do not allow the trait under selection in stratified societies to be identified. One possibility that cannot be excluded is that other types of pleiotropic factors have been selected for, as long as fitness advantages are conferred in a socially stratified context. In any case, social stratification remains the only pivotal identified social variable (i.e., defined above the individual level) associated with MHP in a cross-cultural analysis.

MHP was observed in all highly stratified societies (with at least 5 levels of stratification) of the present sample. As social stratification has occurred independently in various parts of the globe, two scenarios can be proposed about the emergence of MHP. First it is possible that MHP arose independently in those stratified societies. In this case, the life-history traits associated with MHP may not be expected to be the same in different independent societies, depending on the exact nature of the pleiotropic factor that is the target of selection. Some data support this hypothesis, for example, the older brother effect, associated with MHP in Western societies [89]), is not exactly replicated in other stratified societies: in Samoa, MHP is associated with an “older sister” effect [38, 90]. This suggests that the pleiotropic and antagonistic factor expressing MHP is recurrent, although the pleiotropic factor may vary. Alternatively, the factors that favor MHP could have preexisted to the expansion of humans across the globe. In this case, the selection due to the effect of social stratification could have promoted the same preexisting factors that favor MHP. Thus, the life-history traits associated with MHP in the various highly stratified societies should be similar by descent. As an example of supportive data, the frequency of gender atypical behaviors during childhood is reported to be higher in MHP than in heterosexual men (on the basis of recall) in Brazil, Guatemala, the Philippines and the United States of America [91]. This remains an open question, and more data are required.

Another expectation is that MHP may be observed transiently in some non-stratified societies. In the sample obtained from eHRAF, MHP was reported in 4 out of 18 (or 22%) non-stratified societies, namely the Ache, Delaware-Munsee, Iban and Naskapi. There are several possible explanations for the presence of MHP in non-stratified societies. First, the stratification level could fluctuate over time, and some current non-stratified societies could have been more stratified in the recent past [92]. In such cases, MHP could have been selected for during the stratified phase, and selected against but still present while the social system was reshaped without significant stratification. For example, a recent societal collapse (e.g., loss of agriculture) has been proposed for the Ache, when confronted with the expansion of the Guarani people [53]. Second, the proximity of a stratified and dominant society could lead to asymmetrical gene flow and hence migration of the MHP loci in non-stratified societies. In such a case, genes selected for in the context of the stratified society could be found at a relatively high frequency in the non-stratified one, despite the continuous selection against such factors. For example an asymmetrical admixture pattern has been observed between agriculturalist and pygmies populations of central Africa, with a gene flow directed from the stratified group toward the non-stratified one [93]. Whether such a situation contributes to an explanation for the presence of MHP in the four cases above remains to be evaluated. Finally, some misreporting of rare scenarios cannot be excluded. For example, some individuals considered as “hermaphrodites” by the natives were sometimes described as being a “man-woman” (e.g., [94], a classical name for a social third gender. Such a phenomenon is most likely limited, as intersexuality has an estimated prevalence of 0.018% [95], resulting in 1–2 cases every 10,000 individuals.

Social stratification remains the only known social variable significantly influencing the presence of MHP in a given society. This is consistent with the idea that a pleiotropic and antagonist factor is the target of selection in stratified societies and that MHP imposes a fitness cost on male fertility. Whether the selected trait is female fertility (under the hypergyny hypothesis) or another life-history trait has yet to be evaluated, although the selected trait may differ among various independent stratified societies. Data from pristine prehistoric societies (i.e., isolated from the influence of stratified societies) are currently non-existent. Unless other social variables (independent from social stratification) influencing MHP are identified, societies with low levels of stratification are predicted to display, at best, a low level of MHP individuals. Social stratification as a promoting factor of MHP is also consistent with currently available data showing that MHP seems to be absent in wild animals. This is because the type of social stratification displayed in humans has no equivalent in other animals. Human social stratification is defined across generations, and a given individual generally belongs to the same social class during his entire lifetime, and usually reproduces within the same class. The dominance rank in social non-human animals is generally transient (e.g. the tenure of the alpha chimpanzee lasts only few years, [96]) or, if transmitted to the next generation, restricted to one sex (e.g. female ranks among the spotted hyaena and some Cercopithecines species [97, 98])

## Conclusion

Relatively few studies have been performed to understand the origin of MHP, despite a widespread misinformed homophobia, and thus a social need for scientific knowledge on this subject. Each step toward a better understanding of the evolution and spread of MHP among humans would contribute to a constructive social debate.

Here we show that the commonly held view of the virtually universal presence of MHP since prehistoric time in human populations is not confirmed upon review of the cited data. Indeed, the existence of MHP in past times can never be proved or disproved using only archaeological remains: written texts are required to establish that homosexual preference was eventually present, and this information is probably definitively inaccessible for prehistoric (e.g., before written texts) societies. Today, MHP appears to be absent in some societies but present in others; this variability can be partly explained by the level of social stratification. This is consistent with a factor being selected for in a stratified society, despite a pleiotropic cost on functional male fertility (MHP). One possible candidate is a factor increasing female fertility, specifically by increasing the probability that a female marries males from higher social classes when hypergyny is enforced. As stratified societies are relatively recent (generally post-Neolithic), the substantial prevalence of MHP is most likely a recent phenomenon in humans and much remains to be understood.

## Supporting Information

S1 Data Supporting data. (ZIP)

S1 Fig Geographic distribution of the sampled societies (using the eHRAF to assess the level of stratification). Full circles: societies with MHP; empty circles: societies without MHP.
(TIFF)

S1 Table Archaeological data often cited as evidence of MHP in prehistoric societies. (PDF)

S1 Text Supporting information. (RTF)
