# Supplementary figures and images for: Male Homosexual Preference: Where, When, Why?
Source: PLoS One. 2015 Aug 12;10(8):e0134817. doi: 10.1371/journal.pone.0134817 (PMC4534200; doi:10.1371/journal.pone.0134817)

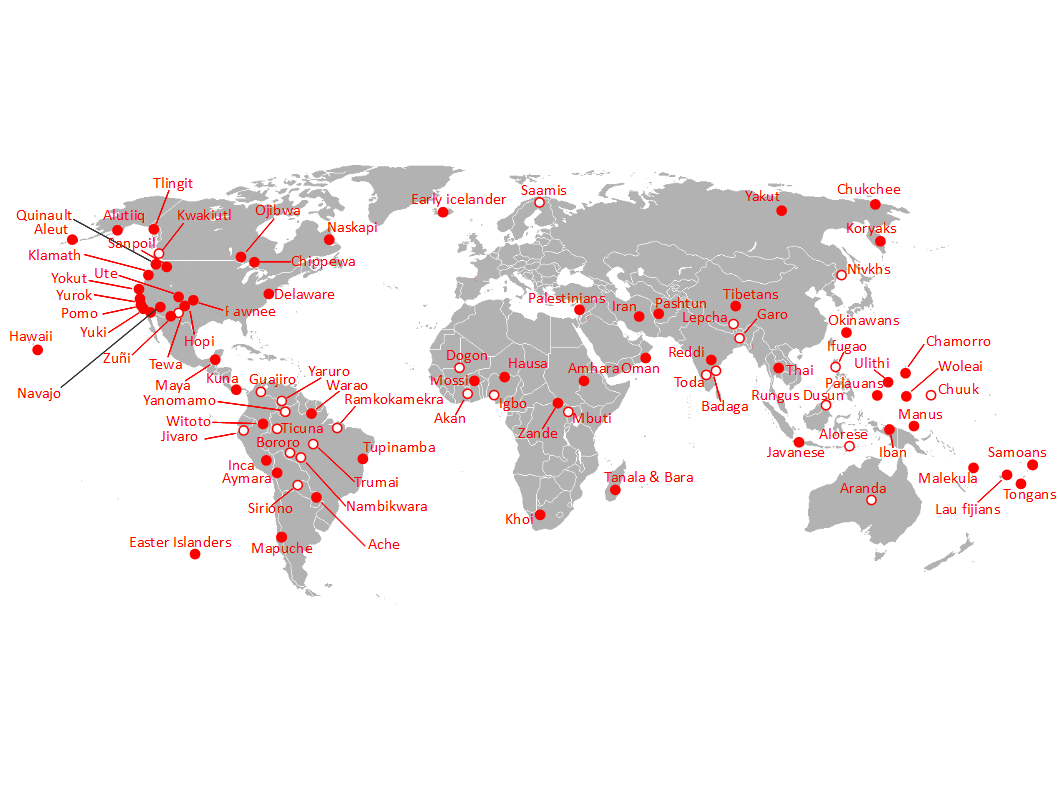

Supplement: S1 Fig — Full circles: societies with MHP; empty circles: societies without MHP. (TIFF) [file pone.0134817.s002.tiff]
