# Supplementary material for: Male Homosexual Preference: Where, When, Why?
Source: PLoS One. 2015 Aug 12;10(8):e0134817. doi: 10.1371/journal.pone.0134817 (PMC4534200; doi:10.1371/journal.pone.0134817)
Supplement: S1 Table — (PDF) [file pone.0134817.s003.pdf]

Country	Ethnic group	Date	Type	Place	Description	Author
Norway	European	Mesolithic	Rock engraving	Bardal Panel	Two of the human figures are locked in "rear-entry" sexual intercourse and have previously been interpreted as being male and female by Hallström (1938). The smaller figure may possibly have breasts but, equally, the two lines here could well be a pair of arms. A series of vertical lines possibly stylised pubic hair, hangs below the lower abdomen. The large headless figure penetrating the smaller one has an outline of his penis carved inside the smaller figure. The position of the penis suggests that penetration is via the anus. Gustave Hallström (1938), however, regards the area of penetration as the vulva. Although the larger figure appears the more dominant, both figures are locked in a harmonious rhythm. There appears to be no sign of any violent sexual penetration -these figures, therefore, could well be deemed as consenting adults. Interestingly, Hallström has also recognised a further two vertical lines directly in front of the smaller figure, which he has interpreted as possibly another human figure. Could this scene, therefore, represent group sexual intercourse, whereby the smaller (central) figure is not only engaged in rear-entry intercourse but also possible fellatio at the same time? [...]	Nash 2001
Spain	European	Mesolithic	Rock engraving	Cuevas de la vieja panel. Albacete	Here, the central figure, a human male (with elegant head-dress), has legs spread between two red deer (which were originally painted as a series of bulls). Another smaller, male figure is positioned between the upper thighs of the central figure. The head of the smaller male is directed to the erect penis of the central (dominant) male – fellatio is being performed.	Nash 2001
Sweden	European	Mesolithic	Rock engraving	Hoghem in Tanum	Nothing marks these figures out as female rather than male. But there are indications that they may both be male :<br>1. In five of the scenes, the "female" figure" has clearly exaggerated calves. Such features are also found on the male figures, and as was noted above, have a strong association with phallic figures generally.<br>2. Within the representational designs of the carvings, pairing as a principle occurs only with definitively male figures – lurs and axes on board ships, paired and confronted warriors or stags – and seldom, if ever, with figures the identity of which is open to doubt [...].<br>It is thus possible that both figures are male – this, of course would require us to suspend our prejudices about what such scenes would then mean, and from my experience it is clear that most archaeologists are unwilling or unable to do so, and will go to extraordinary lengths to hang on to the heterosexual hypothesis... p46	Yates 1993
Peru	Moche	200 BCE-600 CE	At least four vases	Peru	Homosexual acts between males are found on at least four cases (one between a skeletal cadaver and a sleeping male, lying together in bed), each showing consensual anal intercourse. Again, with no ambiguity possible with both sets of male genitalia clearly depicted. There are also many examples where the gender of the penetrated partner is ambiguous, but these are usually interpreted as heterosexual coupling, nonetheless. Homosexuality is also contested by certain authors who deny the existence of such objects [...] It is difficult not only to attribute precise dates and provenance but to assign valid and convincing interpretation and to attach meaning (not only their meaning for us, but their possible meaning for the Moche culture itself) to these objects, since we have no text to interpret them [...] other than brief mentions in the incomplete, fragmented and very biased codices and writings of Spanish missionaries and conquistadors.	Mathieu 2003
Egypt	Egyptian	2400 BCE	Egyptian grave	Necropolis of Saqqara	I agree somewhat with John Baines when he says "Since the embracing and handholding scenes are unique in private tombs, little can be said about their meaning beyond the fact that they express publicly the close involvement of the two men" (Baines 1985:467). The hand-holding and embracing scenes may be unique between men of equal station in private tombs but not for husband and wife. It is when the totality of intimate scenes in the tomb of Niankhkhnum and Khnumhotep are compared to the innovative conjugal figurations of the Fourth, Fifth and Sixth Dynasties of the Old Kingdom that same-sex desire and sentiment must be considered as a probable explanation. Whatever the biological relationship may have been between Niankhkhnum and Khnumhotep, their iconography vocabulary was most closely aligned to that used to portray conjugal sentiment between husband and wife.	Reeder 2000

S1 Table. Archaeological data often cited as evidence of MHP in prehistoric societies.
