# Supplementary material for: Male Homosexual Preference: Where, When, Why?
Source: PLoS One. 2015 Aug 12;10(8):e0134817. doi: 10.1371/journal.pone.0134817 (PMC4534200; doi:10.1371/journal.pone.0134817)
Supplement: S1 Text — (RTF) [file pone.0134817.s004.rtf]

Supplementary informations
1) Class stratification variable - eHRAF
From the excerpts available in the eHRAF database, the level of stratification was first assessed for each society on a scale ranging from 1 to 5, corresponding to the number of classes that could be identified, and then reduced to 4 levels. The limit between two classes been sometimes vague, some societies were classified as intermediary between two categories (e.g., 2.5 classes). For example, bases on the following excerpts: “Tongan society is today a stratified hierarchy with royalty and nobles at the top of the social scale, the common people at the bottom. In some villages, a high chief or noble has his permanent residence. For the rest, the villagers are just Tongans of the commoner class.” from Beaglehole [1], “Tongan society, traditionally, was very hierarchical, there being a structure of ranked nobles and commoners. […] The main social strata of Tongan society consist of the king and his family, the nobles and chiefs, and the commoners” from Bloomfield [2] and “In the former Tongan population there was evidently a still lower element than the commoners, the population (slave, prisoner)” from Gifford [3], five classes were identified for the Tongans of Polynesia, including, kings nobles, local chiefs, commoners and slaves. It could be thus concluded that the Tongan society was highly stratified. The resulting variable corresponding to the number of classes was then reduced to a three levels variable with the following factors: (1) no stratification (between 1 and 1.5 classes), (2) moderately stratified (between 2 and 3.5 classes), and (3) strongly stratified (4 or more classes). 
The societies were first classified by JB. Then, the 74 societies for which the monographs were directly available (using the eHRaF) were classified independently by MR with the same information, but without the identity of the society. The level of agreement between the two ratings was assessed using a weighted Cohen's Kappa, κ = 0.80 [CI: 0.67-0.94], z = 6.98, P < 0.0001, i.e., “almost perfect” following Landis [4; 5].
2) Class stratification variable - EA
The five factors of the "Class stratification" variable of the EA have been merged into three levels: (1) absence of social stratification (factor 1), (2) simple stratification based on wealth or elite (merging factors 2 and 3) and (3) complex stratification (merging factors 4 and 5). Indeed, Generalized Estimating Equation method, as implemented in the ape package [6], does not allow empty classes. Merging levels of stratification allows to address this constraint and provides a simplified coding.
3) Archaeological evidences
The petroglyphs from Tanum, Sweden were analyzed. It consists in stone carving possibly representing a man couple [7]. While frequently cited as evidence of MHP during prehistory, the argument is restricted to a statement, suggesting that interpretation of the stone carving is out of “our prejudices about what such scenes would then mean”, even if the alternative hypothesis of a heterosexual couple cannot be excluded [7]. Indeed, the author states that “Nothing marks these figures out as female rather than male” (p. 45). Thus, the statement that this is a representation of a homosexual couple is not supported by any conclusive evidence. This prehistoric reference cannot therefore be considered as archaeological evidence of the existence of MHP in prehistoric societies.
Evidence based on the ceramics from the Moche culture (200 BCE – 600 CE) from Peru (see Table 1) were studied. More than 800 ceramics have been found representing explicit heterosexual events, with the exception of four of them (~0.5 %) representing anal intercourse between two men. This suggests that same-sex intercourse was conceivable (and probably practiced) for the Moche. However, as highlighted by Mathieu [8], the authenticity of these four ceramics has not been established since no dating has been performed.
The earliest possible evidence of the presence of homosexual preference from the archaeological record is most likely the painting of the mausoleum of Khnumhotep and Niankhkhnum from Ancient Egypt (circa 2400 BCE), interpreted with the help of written texts (thus not a prehistoric case). Khnumhotep and Niankhkhnum were two male manicurists of the king. These two men were buried in the same mausoleum and iconographic vocabulary in the mausoleum portrays them as having mutual conjugal sentiments (e.g., holding hands, directing their gaze into each other's eyes, while in an intimate embrace) [9].
References
1. Beaglehole, E (1941) Pangai: village in Tonga. Wellington, N.Z.: The Polynesian Society.
2. Bloomfield, SF (2002) Illness and cure in Tonga: traditional and modern medical practice. Nuku'alofa Tonga: Vava'u Press.
3. Gifford EW (1929) Tongan society. Honolulu Hawaii: The museum.
4. Landis JR and Koch GG (1977) The measurement of observer agreement for categorical data. biometrics: 159-174.
5. Cohen J (1960) A Coefficient of Agreement for Nominal Scales. Educational and Psychological Measurement 20: 37-46.
6. Paradis E, Claude J and Strimmer K (2004) APE: Analyses of Phylogenetics and Evolution in R language. Bioinformatics 20: 289-290.
7. Yates T (1993) Frameworks for an archaeology of the body. In: T. C., editor editors. Interpretative archaeology. Providence, RI: Berg Publishers. pp. 31-72.
8. Mathieu P (2003) Sex pots: Eroticism in ceramics. Rutgers University Press.
9. Reeder G (2000) Same-sex desire, conjugal constructs, and the tomb of Niankhkhnum and Khnumhotep. World Archaeology 32: 193-208.
